# Physiological culture conditions alter myotube morphology and responses to atrophy treatments: implications for in vitro research on muscle wasting

**DOI:** 10.14814/phy2.13726

**Published:** 2018-06-21

**Authors:** Elodie Archer‐Lahlou, Cathy Lan, R. Thomas Jagoe

**Affiliations:** ^1^ Lady Davis Institute for Medical Research Segal Cancer Centre Jewish General Hospital McGill University Montreal Quebec Canada

**Keywords:** Cancer cachexia, hypoxia, muscle atrophy models, muscle tissue culture

## Abstract

Standard in vitro myotube culture conditions are nonphysiological and there is increasing evidence that this may distort adaptations to both catabolic and anabolic stimuli and hamper preclinical research into mechanisms and treatments for muscle atrophy in cancer and other chronic diseases. We tested a new model of myotube culture which mimics more accurately the basal conditions for muscle tissue in patients with chronic disease, such as cancer. Myotubes derived from C2C12 myoblasts, cultured under the modified conditions were thinner, more numerous, with more uniform morphology and an increased proportion of mature myotubes. Furthermore, modified conditions led to increased expression of mir‐210‐3p, genes related to slow‐twitch, oxidative phenotype and resistance to commonly used experimental atrophy‐inducing treatments. However, treatment with a combination of drugs used in anti‐cancer treatment (doxorubicin and dexamethasone) under the modified culture conditions did lead to myotube atrophy which was only partially prevented by co‐administration of curcumin. The results underline the importance and potential advantages of using physiological conditions for in vivo experiments investigating mechanisms of muscle atrophy and especially for preclinical screening of therapies for cancer‐related muscle wasting.

## Introduction

Cancer‐associated muscle wasting is a cardinal feature of cancer cachexia (Fearon et al. 2011) and is strongly associated with reduced tolerance of anti‐cancer treatments (Prado et al. 2009) and poor survival (Prado et al. 2008). Recent controlled clinical studies with three different agents (Espindolol, Enobosarm, and Anamorelin) have demonstrated modest benefit in improving lean body mass (Dobs et al. 2013; Stewart Coats et al. 2016; Temel et al. 2016). However, even these newer drugs were not sufficiently active in isolation to achieve the desired improvements in both performance status and physical function. Thus, despite many years of research in this field, further studies are needed to find treatments which both stop or reverse cancer‐related muscle loss and enhance muscle function.

Performing clinical trials in cancer patients with muscle wasting is very challenging and high drop‐out rates are common (Macdonald 2007). Well‐designed preclinical models still have a vital role to play in screening different potential myoprotective treatments and guiding the selection of the best candidates for more detailed clinical testing. Experiments using cultured myotubes are a key step in investigating the direct effects of catabolic agents and to determine the protective effect of anti‐muscle atrophy drugs and nutraceuticals. Typically, experimental outcomes employ measurements of myotube morphology (e.g., fiber diameter) and expression levels of anabolic and catabolic genes to determine responses. Unfortunately, standard conditions for myotube culture, including studies of adaptations to catabolic and anabolic stimuli, are far removed from those found under physiological conditions, despite mounting evidence that using physiologically relevant environmental conditions, including oxygen tension, for in vitro models, can have wide‐ranging impact on cellular phenotypes and molecular adaptations (Keeley et al. 2017).

Several authors have tested a variety of approaches to improve myotube growth, differentiation and survival, with some success. These have included the use of two‐ (Langen et al. 2003; Cooper et al. 2004; DeQuach et al. 2010) or three‐dimensional (Kroehne et al. 2008; Mozetic et al. 2017) biomolecular or inert supports, and use of electrical stimulation (Thelen et al. 1997; Bayol et al. 2005; Boonen et al. 2011; van der Schaft et al. 2013). However, such experimental conditions have not been widely adopted by other research groups, nor have there been attempts to test myotube culture conditions that combine several features of the in vivo milieu to study disease‐related muscle atrophy.

Current standard myotube culture conditions use isolated myotubes under supra‐physiological oxygen tension growing in culture medium containing serum growth factors. However, prior data suggest that reduced oxygen tension (Green et al. 1989), denervation (Sacheck et al. 2007) and higher circulating levels of proinflammatory cytokines (Guttridge et al. 2000; Langen et al. 2001) promote reduction in muscle mass or altered myoblast maturation. Thus, we developed and tested a new model of myotube culture incorporating low doses of proinflammatory cytokines, physiological levels of oxygen and low‐frequency external electrical stimulation to try to reproduce important environmental factors present in vivo in patients with cancer and chronic diseases that are relevant to muscle tissue responses (chronic disease modified: CDmod). To test the hypothesis that the modified culture conditions would alter myotube growth and response to atrophy treatments, we compared myotube growth under CDmod and standard (STD) conditions. In addition, atrophy responses of myotubes, after treatment with dexamethasone (DEX) under CDmod and STD conditions were compared.

It is now clear that cancer‐related muscle atrophy may result from both metabolic changes due to the cancer and direct catabolic effects of several anti‐cancer drugs on muscle tissue, for example, (Antoun et al. 2010). Indeed, combined chemotherapy treatment for cancer is implicated as a cause for muscle loss and dysfunction in cancer survivors (Scheede‐Bergdahl and Jagoe 2013), and we recently showed that combined treatment with dexamethasone and doxorubicin (DD) causes persistent reduced muscle mass and mitochondrial dysfunction in non‐cancer bearing mice (Gouspillou et al. 2015). Using the modified culture conditions, we measured the effect of DD treatment on myotube morphology and gene expression and went on to test the impact of curcumin as a potential myoprotective agent.

## Material and Methods

### Cell culture

Mouse C2C12 skeletal myoblasts were purchased from ATCC. C2C12 myoblasts were grown in high‐glucose DMEM (Gibco) supplemented with 10% FBS (Invitrogen) and used at low passage (less than 10) for all experiments. Unless otherwise stated myoblasts were seeded on 0.15% gelatin‐coated (Sigma #G9391) 6‐well plates (6 × 10^4^ cells per well) and grown to 80% confluence then transferred to differentiation medium containing 2% horse serum (Invitrogen). For all experiments myotubes were cultured under standard (STD) culture conditions (21% O2, 5% CO2 in a humidified incubator at 37°C) until day 5. On day 5 postdifferentiation, myotubes either remained in STD conditions or were transferred to modified culture conditions (CDmod) described below. Preliminary studies with the C2C12 myoblasts were performed under both STD and CDmod conditions which established that myotubes were healthy and adherent until around day 9 postdifferentiation, after which they began to senesce and detach. Additional experimental treatments were started on the morning of day 5 postdifferentiation, and continued for 3 days. In all experiments, the culture medium and any additional soluble drug treatments were renewed every 24 h. CDmod conditions had the following additional features: (1) physiological tissue PO2 (4 kPa = 4%O2) (Richardson et al. 1995), using a humidified incubator (Thermo Forma, Series II) combined with nitrogen displacement to achieve desired oxygen tension; (2) imposed low‐frequency contraction using a purpose‐built in‐plate stimulation rig (Marotta et al. 2004) at 1 Hz for 18 h followed by 0.1 Hz for 6 h using a biphasic (5 msec) square‐wave signal generator at 20 V. The pattern of electrical stimulation was chosen to reflect diurnal variation in skeletal muscle activation whilst avoiding over‐heating or electroporation in plate; (3) clinically relevant levels of proinflammatory cytokines by daily addition of TNF‐*α* (20 pg/mL; R&D systems) and IL‐6 (100 pg/mL; R&D systems) (Martín et al. 1999; Bossola et al. 2000) diluted in differentiation medium. All morphometric and molecular analyses were performed at day 8 postdifferentiation.

### Experimental treatments

(1) Dexamethasone treatment (Dex) (1 *μ*mol/L; Sigma D 2915) dissolved in sterile water; (2) Doxorubicin, supplied as 2 mg/mL in sterile water by Jewish General Hospital pharmacy, administered at a final concentration of 0.2 *μ*mol/L in differentiation medium; (3) Curcumin kindly supplied in pure crystal form by Dr Jian Hui Wu, and administered at 0.1 *μ*mol/L in differentiation medium. Nontreated control plates were not given additional sterile water or differentiation medium vehicule.

### Protein and DNA extraction

On day 8 postdifferentiation, myotubes were washed with PBS and DNA was extracted using DNeasy DNA blood and tissue kit (QIAGEN). For protein extraction, cells were incubated in 220 *μ*L lysis buffer (M‐PER Mammalian Protein extraction reagent (ThermoScientific) with PhosphoSTOP phosphatase inhibitor cocktail and Complete Mini Protease inhibitor Cocktail, Roche) for 5 min at room temperature. The cell lysate was then centrifuge at 12,000 *g*, 4°C, for 20 min and the supernatant was used for total protein concentration analysis using the BCA assay (Pierce).

### Western blotting and antibodies

Equal amounts of protein (30 *μ*g) were separated by SDS‐PAGE and transferred to a nitrocellulose membrane (BioRad). The membrane was blocked for 1 h at room temperature with 5% milk in TBS‐Tween (TBS‐T) and then incubated with the primary antibody in 5% BSA in TBS‐T (for p‐AKT, 1% BSA in TBS‐T) overnight at 4°C. After washing in TBS‐T, the membrane was incubated with a horseradish peroxidase‐conjugated secondary antibody (GE Healthcare) and visualized using enhanced chemiluminescence reagents (Pierce ECL, ThermoScientific). Bands from each blot were quantified via densitometry using Gene Snap and Gene Tool image acquisition and analysis software (Syngene) and normalized to the loading control (‐tubulin). The antibodies used include mouse monoclonal anti‐myosin skeletal, slow (MyHCs M8421) and fast (MyHC‐f M4276) (1:1000; Sigma); rabbit polyclonal anti‐AMPK (2532) and anti‐phospho‐AMPK (Thr172; 2531) (1:1000; Cell signaling); rabbit polyclonal anti‐myoglobin (1:2000; Abcam ab77232); rabbit polyclonal anti‐AKT (9272) and anti‐phospho‐AKT (ser473; 9271) (1:1000; Cell signaling); mouse monoclonal anti‐tubulin (1:8000; Sigma T 6074); anti‐mouse and anti‐rabbit HRP‐conjugated secondary antibodies (1:5000, GE Healthcare).

### Jenner‐Giemsa staining and fusion index scoring

On day 8 postdifferentiation, myotubes were washed with PBS, fixed with 100% methanol for 10 min at room temperature, and then washed again with PBS. If not stained immediately, 6‐well plates containing fixed cells were stored at 4°C. For Jenner‐Giemsa staining cells were incubated with 1 mL of Harleco® Jenner stain solution (EMD Millipore) for 5 min at room temperature, followed by three washes with distilled water. Cells were then incubated with 1 mL of Harleco® Giemsa stain solution (EMD Millipore) diluted 1/25 in 1 mmol/L sodium phosphate buffer (pH 6.8) for 10 min at room temperature. Cells were finally washed four times with distilled water. Cells were visualized and photographed with a 20X objective, using a digital camera (Canon PC1089) adapted to an inverted microscope (Zeiss, Axiovert 40 CLF). Six images per well were captured randomly in three wells for each condition. Protein‐rich myotubes can be identified by a dark purple color, whereas nuclei stain pink, and in each field, the number of nuclei incorporated in myotubes, the total number of nuclei and the number of myotubes were scored. Fusion index (FI) was calculated as the percentage of total nuclei incorporated in myotubes. Maturation index (MI) was calculated as the mean number of nuclei per myotube. The data to calculate FI and MI index was collected by a single observer (EA).

### Myotube morphology analysis and counting

Myotubes were visualized and photographed with a 10X objective on a Nikon CKX41 inverted microscope and an Infinity‐1 digital camera (Lumenera Corporation) on day 8 postdifferentiation for all experimental and control conditions. Five images per well were captured randomly in six wells for each condition. Measurements were made using ImageJ software (Rasband 1997). Two orthogonal lines were drawn through the centre of each image and myotube diameter was measured where each line transected the myotube. The morphometric analysis was performed by an independent observer who was blinded to the culture conditions.

### RNA isolation and gene expression using quantitative RT‐PCR

Total RNA extraction from myotubes was performed using QIAzol Lysis reagent and RNeasy Mini Kit with on‐column DNase digestion (QIAGEN). RNA concentration was determined with NanoDrop ND2000 spectrophotometer (Thermo Scientific). Total RNA was reverse transcribed using SuperscriptTM III First‐Strand Synthesis SuperMix for qRT‐PCR (Invitrogen). The relative mRNA levels were determined by real‐time PCR using Taqman method in a 96‐well format on a Mastercycler ep Realplex (Eppendorf) for mouse muscle atrophy F‐box protein 32 (Atrogin1/Fbxo32), Cathepsin‐L (Ctsl), Insulin‐like growth factor‐1 (Igf1), Muscle‐specific RING finger protein 1 (MuRF1/Trim63) and Peroxisome proliferator‐activated receptor gamma coactivator 1‐alpha (Pgc1), using the following Taqman probes: Mm499523_m1, Mm00515597_m1, Mm00439560_m1, Mm01185221_m1, and Mm01208835_m1, respectively. After testing a number of different potential candidates Mouse 18S (hs99999901_s1), Cyclophilin (Mm02342429_g1), and RPLPO (Mm01974474_gH) were adopted as reference genes. Triplicates of each of three experimental replicates for each experimental condition were performed. Amplification efficiencies for each probe were empirically determined and relative quantification was calculated using the Pfaffl method adjusted to normalize for all three reference genes (Pfaffl et al. 2002).

### miRNA expression profiling

Two *μ*g total RNA from each experimental sample was covalently 3′‐labeled with a single Hy3 (test) or Hy5 (reference) fluorophore per molecule, and co‐hybridized using a fully automated hybridization station (Tecan HS400Pro) to microarrays prespotted in quadruplicate with capture probes for approximately 2000 mouse, rat, human sequences from miRBase (v.11 – v.16) (Hi‐Power Labeling kit, Spike‐in miRNA controls and miRCURY LNA microRNA arrays v.11, 5th and 6th generation, Exiqon, Woburn MA). To determine the variance for expression for each miRNA due to differential characteristics of the two fluorophores, the same image analysis steps were used for four “self‐self” experiments in which a single RNA sample was split between two aliquots, labeled with either Hy3 or Hy5 and hybridized to the same microarray. After hybridization slides were scanned (Model G2505B, Agilent Technologies, Santa Clara, CA) and the two‐color images obtained were analyzed using Spot v3.1 (CSIRO, NSW, Australia) package in R v2.12 (R Development Core Team, 2012). Median signal intensity from each spot was corrected using subtraction of local background and within‐array normalization of the log2 ratio of the corrected spot signal from each channel was performed using the loess method in FlexArray v1.6.1 (Blazejczyk et al. 2007). Additional experimental and complete expression data for these experiments is available at NCBI Gene Expression Omnibus under reference GSE81138 (http://www.ncbi.nlm.nih.gov/geo/query/acc.cgi?acc=GSE81138).

### Fluorescence imaging of myotubes with Mitotracker Red

It was not possible to perform fluorescence imaging of myotubes on the 6‐well plates within the electrical stimulator rig, thus to obtain these images, C2C12 were cultured on plastic coverslips precoated with 0.15% gelatin and placed within the 6‐well plates. Myoblasts were seeded on plastic coverslips at 6 × 10^4^ cells per well, grown to 80% confluence, and then transferred to differentiation medium containing 2% horse serum. On day 5 postdifferentiation, myotubes were transferred to modified culture conditions, under 4% O2 and with daily TNF‐*α* and IL‐6 as described above, but without external low frequency electrical stimulation (CDmod(s) conditions). Any additional experimental treatments were also started on the morning of day 5 postdifferentiation, and continued for 3 days. On day 8 postdifferentiation, cells were incubated at 37°C for 25 min with a Mitotracker Red CMXRos dye (Molecular Probes; 100 mmol/L), washed once in warm DM and twice with PBS and then fixed for 20 min in 4% PFA. The fixed cells on plastic coverslip were mounted on a slide using Immumount (Thermo Scientific) and visualized using a Leica DM‐LB2 microscope.

### Statistical analysis

Raw data for protein yield and myotube dimensions was normalized (%) to the value for myotubes from the same experimental run, cultured under STD conditions to account for differences due to seeding density and growth rates. Data is expressed as mean (SD), mean (SEM) or box‐plots with inter‐quartile range as indicated. Significance testing was performed using students *t*‐tests paired by experimental run using (R Development Core Team, 2012). For miRNA experiments the mean normalized log2 expression ratios for each miRNA calculated. No result was recorded if less than half of experimental replicates yielded analyzable results. Differential expression of CDmod versus STD was first determined using the Significance Analysis of Microarrays (SAM) v4.0 add‐in for Microsoft Excel(Tusher et al. 2001) on array‐centred data from each of six experiments using a threshold false discovery rate of <1. For those genes identified using the SAM algorithm, a Student's *t*‐test was performed to compare gene‐specific mean expression ratio in experimental samples, with mean ratio for same gene derived from self‐self hybridization experiments. A *t*‐test significance level was set as *P* < 0.01 to achieve an expected false positive rate of less than 1. Only those genes which were positively identified as differentially expressed using both these methods, were used in further analysis.

## Results

### Culturing myotubes under CDmod conditions changes myotube growth and morphology

**Figure 1:**
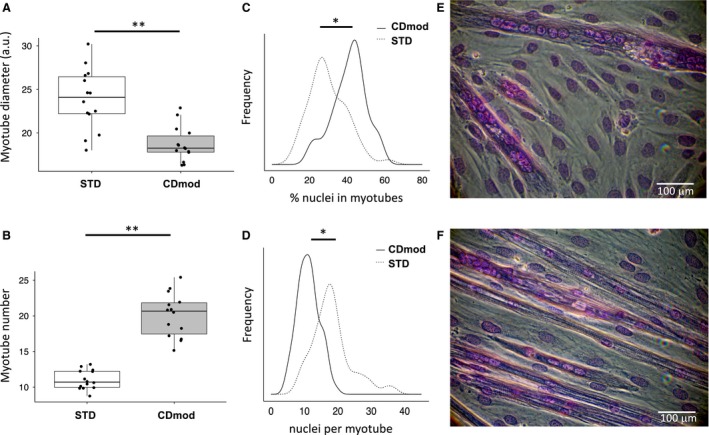
Morphological changes observed in myotubes cultured under CDmod conditions. (A) Boxplots of myotube diameter (arbitrary units) and (B) number of myotubes per field, in CDmod and STD conditions (*N* = 14). (C) Density plots of pooled data for proportion (%) total nuclei found in myotubes in CDmod and STD conditions and (D) Density plots of the number of myonuclei found in each formed myotube (data from 3 independent experiments). (E and F) are representative pictures of myotubes under STD and CDmod conditions, respectively. Culture under CDmod conditions leads to narrower, more abundant myotubes with lower proportion of unfused reserve myoblasts and higher fusion index. *P < 0.05, **P < 0.001 comparing CDmod and STD using paired *t*‐test.

To evaluate the effect of CDmod conditions on myotube growth and morphology, we compared mature C2C12 myotube cultures under standard (STD) versus modified (CDmod) conditions. CDmod conditions lead to 21% reduction in myotubes diameter but twice as many myotubes per plate compared to STD conditions (Fig. 1A and B, Table 1). In addition, fusion of myoblasts to form multinucleate myotubes was significantly improved under CDmod conditions compared to STD. The proportion of nuclei incorporated into myofibers (fusion index) was significantly increased in CDmod conditions, but the average number of nuclei in multinucleated myofibers (maturation index) was lower compared to STD (Fig. 1C and D, Table 1). Moreover, the variance in number of nuclei per myotube was also significantly lower in CDmod conditions (pooled variance CDmod vs. STD: 10.3 vs. 35.3, *F*‐test *P* < 0.001) (Fig. 1D). This is consistent with CDmod conditions reducing the tendency of myotubes to form myosacs with localized accumulations of multiple nuclei that is frequently observed in STD conditions (Fig. 1E and F). Thus overall, fusion of myoblasts was more efficient under CDmod conditions and the mature myotubes formed were more numerous, aligned more regularly and with a more reproducible morphology (Fig. 1E and F).

**Table 1:** Morphological and molecular adaptations in myotubes cultured under standard (STD) and modified (CDmod) conditions

Culture conditions	STD	CDmod	Experimental replicates (*N*)
Myotube diameter (a.u.)	23.9 (3.5)	18.7 (2.0)**	14
Myotube number (per field)	11.0 (1.3)	20.1 (3.1)**	14
Total number of nuclei (per field)	388.0 (30.9)	331.9 (37.9)*	3
% of nuclei in myofibers (fusion index)	29.8 (4.0)	40.9 (3.8)*	3
Nuclei per fiber (maturation index)	18.3 (1.5)	11.2 (0.5)*	3
Total protein (mg)	908.6 (144.3)	892.2 (98.2)	6
Relative protein yield		0.99 (0.1)	6
Relative protein expression			
MyHC‐s		2.0 (1.1)*	9
MyHC‐f		1.0 (0.1)	6
Mb		2.5 (1.4)*	9
Akt		0.9 (0.3)	9
pAkt		0.5 (0.4)*	9
pAkt/Akt		0.6 (0.4)*	9
AMPK		1.3 (0.4)*	9
pAMPK		1.4 (0.9)	9
pAMPK/AMPK		1.1 (0.4)	9
Relative mRNA levels			
Pgc1		1.3 (0.4)*	7
Igf1		0.3 (0.1)**	7
MuRF1/Trim63		1.1 (0.3)	7
Atrogin1/Fbxo32		1.3 (0.1)**	7
Ctsl		1.2 (0.2)*	7

Measurements performed at day 8 postdifferentiation for myotubes cultured under STD versus CDmod conditions (O2 4 kPa, low frequency electrical stimulation, TNF‐*α* 20 pg/mL and IL‐6 100 pg/mL). Data are mean (SD). Paired *t*‐tests (by experimental replicate for raw data) and one sample *t*‐test (relative expression data) compare CDmod versus STD. *P < 0.05, **P < 0.001.

### CDmod culture conditions increase expression of selected slow‐twitch oxidative muscle proteins

**Figure 2:**
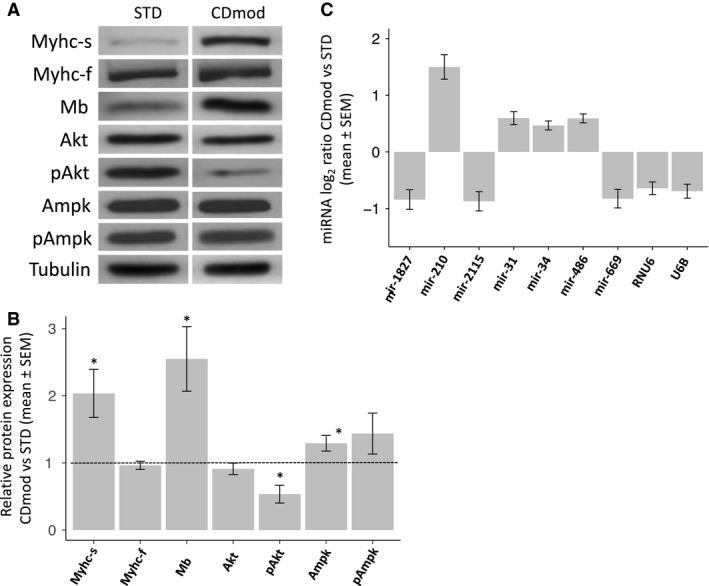
Gene expression changes observed in myotubes cultured under CDmod conditions. (A) Representative immunoblots comparing expression levels of selected proteins in mature myotubes under STD and CDmod conditions. (B) Quantitative analysis of same proteins as A (minimum 3 experimental replicates). *P < 0.05, **P < 0.001 compared to expression in myotubes under STD conditions. (C) Relative levels (log2 scale) of the six confirmed differentially expressed miRNAs or small RNA species under CDmod conditions.

Myotubes cultured under CDmod conditions had the same total protein content (Table 1). However, CDmod conditions induced modestly increased levels of Pgc1 mRNA and increased expression of Myhc‐s and Mb protein levels (Fig. 2A and B, Table 1).

### Myotubes cultured under CDmod conditions have reduced expression of anabolic and increased expression of some catabolic pathway components

CDmod conditions induced some proteolysis‐related genes and reduced expression of some anabolic signaling molecules. Thus, mRNA levels for Fbxo32 and Ctsl were increased in CDmod conditions, but mRNA levels for another proteolysis‐related gene, MuRF‐1, were unchanged (Table 1). In addition, mRNA levels for Igf1 were markedly reduced in CDmod conditions. At the protein level, no difference in pAmpk or pAmpk/total Ampk ratio was observed, but CDmod conditions did cause reduced pAkt and pAkt/total Akt ratio (Table 1, Fig. 2A and B).

### CDmod culture conditions lead to increased expression of mir‐210‐3p

In parallel experiments, profiling of miRNA and related small RNA species was performed to compare myotubes cultured under STD and CDmod conditions (see Data S1 for methods). RNA from six independent experiments was hybridized to miRNA‐specific microarrays. Of 71 miRNAs identified as potentially differentially expressed in CDmod conditions, 6 miRNA/small RNAs were confirmed as significantly differentially expressed and 36 miRNAs were confirmed as not differentially expressed (a further 29 remained potentially differentially expressed but could not be corroborated due to incomplete data from the corresponding probes in the self‐self hybridization experiments). Of the six confirmed candidate differentially expressed miRNAs, only one was differentially expressed more than two‐fold, namely mir‐210‐3p, which was increased 2.8‐fold (1.5 on log_2_ scale) in CDmod conditions (*P* = 0.001) (Fig. 2C).

### Dexamethasone treatment in CDmod conditions promotes the slow‐twitch, oxidative phenotype but does not cause atrophy

**Figure 3:**
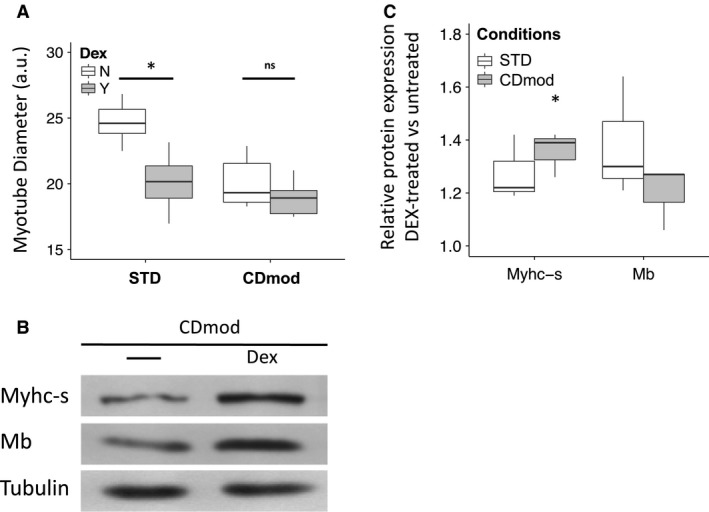
CDmod conditions alter myotube responses to atrophy‐inducing treatments. (A) Boxplots of myotube diameter showing that Dex treatment under STD but not CDmod conditions leads to myotube atrophy. (B and C) Dex treatment under CDmod but not STD conditions, induces increased expression of Myhc‐s. In C, protein expression is expressed relative to that in untreated myotubes cultured in the same conditions. 3 experimental replicates for each comparison. Comparing untreated myotubes under the same culture conditions: *P < 0.05, n.s. = no significant.

We investigated whether the use of CDmod conditions leads to important alterations in the responses of myotubes to experimental atrophy‐inducing treatment. Dex treatment in STD conditions causes a 19% reduction in myotube diameter (*P* < 0.001) and 15% reduction in protein yield (*P* = 0.04) (Table 2, Fig. 3A). However, myotubes cultured under CDmod conditions do not atrophy with Dex treatment alone as myotube diameter and total protein yield were unchanged (Table 2, Fig. 3A). Though Dex treatment was the experimental model studied in detail here, additional parallel experiments using other experimental atrophy treatments showed CDmod conditions yielded resistance to atrophy (smaller reduction in myotube diameter and protein yield) with serum withdrawal and TNF‐related weak inducer of apoptosis (TWEAK) treatment (See Data S1). Though Dex treatment did not cause atrophy under CDmod conditions, a significant increase in Myhc‐s protein was observed (Table 2, Fig. 3B and C). Transcriptional changes induced by Dex treatment were similar in STD and CDmod conditions with up‐regulation of mRNAs for Pgc1, MuRF1 and Fbxo32 and down‐regulation of Igf1 (Table 2). Interestingly, Dex treatment in CDmod conditions was not associated with any further upregulation of mir‐210, as levels of this miRNA remained similar to those seen in untreated myotubes under CDmod conditions (mean(SD) difference 8(2)%, *P* = 0.18). Together these data suggest Dex treatment under CDmod conditions induces adaptive maturation of myotubes rather than a simple atrophy response.

**Table 2:** Differential impact of Dex treatment on myotubes under standard (STD) and modified (CDmod) conditions

Culture conditions	STD		CDmod	
Treatment	Untreated	Dex	Untreated	Dex
Myotube diameter (a.u.)	24.7(1.6)	20.1(2.2)	20.1(2.0)	18.9 (1.4)
Relative diameter		0.8(0.1)**		1.0(0.1)
Total protein (mg)	934.2(191.2)	785.6(107.6)	935.1(115.6)	831.5(76.1)
Relative protein yield		0.85(0.1)*		0.89(0.1)
Relative protein expression				
MyHC‐s		1.3 (0.1)		1.4 (0.1)*
MyHC‐f		1.0 (0.1)		1.0 (0.1)
Mb		1.4 (0.2)		1.2 (0.1)
Akt		1.0 (0.3)		1.1 (0.1)
pAkt		1.0 (0.3)		1.0 (0.4)
AMPK		1.1 (0.1)		0.9 (0.1)
pAMPK		1.0 (0.0)		0.9 (0.1)
Relative mRNA levels				
Pgc1		2.7 (0.8)*		2.6 (0.3)*
Igf1		0.6 (0.1)**		0.4 (0.1)*
MuRF1/Trim63		1.6 (0.3)*		1.9 (0.3)*
Atrogin1/Fbxo32		1.9 (0.2)**		1.6 (0.3)*
Ctsl		1.2 (0.0)*		1.0 (0.0)

Morphological features and relative gene expression at day 8 postdifferentiation for myotubes cultured under STD or CDmod conditions (O2 4 kPa, low frequency electrical stimulation, TNF‐*α* 20 pg/mL and IL‐6 100 pg/mL) and treated with dexamethasone (Dex, 1 *μ*mol/L). Data are mean (SD). Relative expression versus untreated myotubes cultured under same conditions. One sample *t*‐test used to compare values for Dex versus untreated for between 3 and 6 independent experimental replicates for each comparison *P < 0.05, **P < 0.001.

### Combined treatment with dexamethasone and doxorubicin causes myotube atrophy under CDmod conditions which is partially prevented by cotreatment with Curcumin

**Figure 4:**
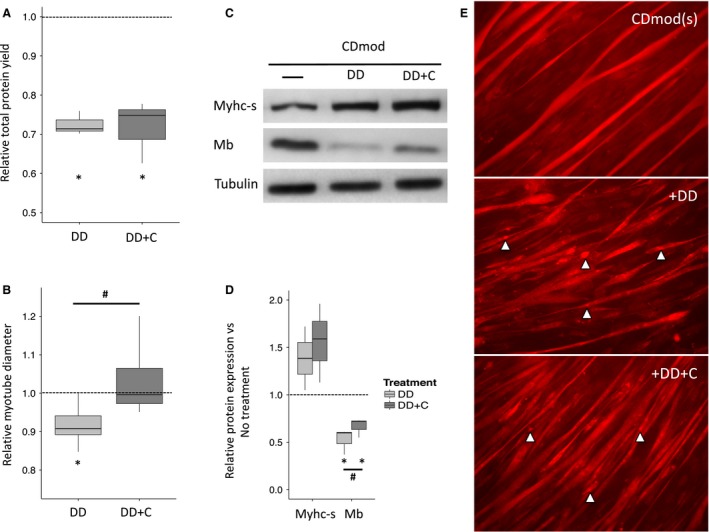
Combined doxorubicin and dexamethasone treatment causes atrophy under CDmod conditions and co‐administration of curcumin has a partial protective effect. (A) Boxplots of showing that myotubes treated with doxorubicin and dexamethasone (DD) with or without curcumin (DD+C) have reduced protein yield. (B) Curcumin co‐administration (DD+C) protects against the DD‐induced reduction in myotube diameter. (C and D) DD treatment leads to significant reduction in Mb expression which is partially rescued by curcumin coadministration. *P < 0.05 compared with untreated myotubes under the same culture conditions, ^#^ *P* < 0.05 comparing DD‐treated and DD+C‐treated myotubes. 3 experimental replicates for each comparison. (E) Qualitative assessment using mitochondrial‐targeted fluorescence imaging showed uniform myotube outline and homogeneous distribution of mitochondrial straining (CDmod (s)). In contrast DD‐treated myotubes (+DD) had variable, thinner diameters with inhomogeneous distribution of mitochondrial staining and prominent highly staining granules (Δ). Coadministration of curcumin (+DD+C) resulted in some improvements in the morphology of myotubes and reduction in number and size of granules (Δ).

In contrast to effects of Dex treatment alone, myotubes cultured under CDmod conditions did atrophy when treated with a combination of Dex and 0.2 *μ*mol/L doxorubicin (DD). DD treatment caused reduced protein yield and myotube diameter (Fig. 4A and B; Table 3), and a significant reduction in Mb (Fig. 4C and D; Table 3). Cotreatment with Curcumin (0.1 *μ*mol/L) partially, but not completely, prevented the changes induced by DD, for example, Curcumin treatment prevented the DD‐induced reduction in myotube diameter (DD vs. DD+C, *P* = 0.02, Fig. 4B) and partially protected against the suppression of Mb expression induced by DD (DD vs. DD+C, *P* = 0.03, Fig. 4D).

**Table 3:** Effects of doxorubicin and dexamethasone (DD) treatment on myotubes cultured under modified (CDmod) conditions

Culture conditions	CDmod		
Treatment	Untreated	DD	DD+C
Myotube diameter (a.u.)	17.9 (2.0)	16.4 (2.0)	18.5 (2.5)
Relative diameter		0.9 (0.1)*	1.0 (0.1)
Total protein (mg)	1090.9 (162.8)	794.3 (153.5)	788.3 (190.1)
Relative protein yield		0.7 (0.0)*	0.7 (0.1)*
Relative protein expression			
MyHC‐s		1.8 (0.7)	1.6 (0.4)
MyHC‐f		1.0 (0.1)	0.9 (0.3)
Mb		0.5 (0.1)*	0.7 (0.1)*
Akt		1.0 (0.1)	1.2 (0.2)
pAkt		1.0 (0.2)	0.9 (0.4)
AMPK		1.0 (0.0)	1.0 (0.0)
pAMPK		0.8 (0.1)	0.6 (0.2)

Morphological features and relative protein expression at day 8 postdifferentiation for myotubes cultured under CDmod conditions (O2 4 kPa, low frequency electrical stimulation, TNF‐*α* 20 pg/mL and IL‐6 100 pg/mL) and treated with DD (dexamethasone, 1 *μ*mol/L and doxorubicin 0.2* μ*mol/L) or DD+C (DD+curcumin 0.1* μ*mol/L). Data are mean (SD). Relative expression versus untreated myotubes. One sample *t*‐test used to compare DD and DD+C versus untreated for between 3 and 6 independent experimental replicates for each comparison *P < 0.05, **P < 0.001.

Standard histochemical staining methods were used for quantitative analysis of myotube diameter. In addition, preliminary studies using low‐resolution, mitochondria‐targeted fluorescence imaging (Mitotracker Red) were performed for qualitative analysis of changes in the myotube mitochondrial network. The results recapitulated the formal morphometric analysis. DD treatment caused disruption of myotube morphology with thinner, irregular myotubes and accumulation of discrete fluorescence‐staining granules indicating disruption of the myotube mitochondrial network (Fig. 4E: CDmod(s) vs. DD). Curcumin cotreatment partially protected against the DD‐induced reduction in myotube diameter and granule formation (Fig. 4E: DD vs. DD+C).

## Discussion

The primary purpose of this study was to confirm whether replicating a combination of key features of the usual extracellular milieu of muscle tissue in patients with cancer (and other chronic conditions), had an impact on the morphology and responses of cultured myotubes. We found that under CDmod conditions, myotubes were about 21% thinner but nearly twice as numerous, with more regular morphology and with higher fusion index, than under STD conditions. In addition, culturing myotubes under CDmod conditions led to increased expression of certain muscle‐specific proteins associated with slow‐oxidative myofiber phenotype. Myotubes cultured under CDmod conditions were not actively atrophying and had the same total protein yield as myotubes under STD conditions. CDmod conditions did not lead to energy depletion (no reduction in pAmpk), however, myotubes under CDmod conditions did have reduced expression of key components of the major protein anabolic signaling axis (Igf1, pAkt,) and increased expression of selected catabolic genes.

The parameters for conditions used in these studies were deliberately chosen to maximize relevance to mature muscle tissue in adults with advanced cancer or chronic disease. The adjusted culture conditions were chosen from first principles and remained stable during the experiments namely: physiological oxygen tension, electrically stimulated contraction and addition of clinically relevant (pg/mL) concentrations of key pro inflammatory cytokines (Martín et al. 1999; Bossola et al. 2000). Finally, experiments were continued for 3 days (days 5–8 postdifferentiation) when myotubes are mature and healthy but prior to onset of senescence and death (day 9 postdifferentiation onwards), and comparisons between treatments were only made at day 8 postdifferentiation. Much prior research in muscle cell culture related to cachexia has focused exclusively on short term responses to different treatments and particularly disruptions which affect myogenesis and early differentiation (Guttridge et al. 2000; Langen et al. 2001). In contrast, we focused on longer term adaptations in mature myotubes, as mature multinucleate myofibers make up the vast majority of muscle mass.

The results presented offer further evidence that including physiologically relevant culture conditions has the potential to modify cellular responses in important ways. However, we accept that there are disadvantages to employing CDmod conditions for studies of myotubes as they are more complex, time‐consuming and expensive than usual STD culture conditions. The potential advantage in using modified myotube culture conditions are likely highest in specific clinically focused projects. These include studies to understanding mechanisms of (and test counter‐measures for) muscle wasting in chronic disease such as cancer. We also accept that there are many unanswered questions about the relative contribution of each of the environmental factors we included in our model. It is likely that other aspects of the model may not have been optimized, for example, the addition of other potentially relevant circulating factors such as growth differentiation factor‐15 (Lerner et al. 2015) or using different patterns of electrical stimulation (Sciancalepore et al. 2015). These details are beyond the scope of this report and can be clarified or developed in future experiments.

Maintaining levels of oxygen (4 kPa) that reflect those found in capillaries supplying muscle tissue (Richardson et al. 1995), is a prominent feature of the CDmod conditions. The 21% reduction on myotube diameter observed under CDmod conditions is very similar to the 23% reduction in myotube diameter reported in engineered muscle fibers cultured at 5 kPa (Martin et al. 2017) and consistent with literature that chronic hypoxia (e.g., high altitude) leads to reductions in cross‐sectional area of muscle fibers in humans, for example, (Green et al. 1989). Hypoxia‐inducible factor‐1a (Hif1a) is constitutively expressed in mature fast‐twitch muscle fibers (Pisani and Dechesne 2005) and induced in other cells after short‐term exposure to more severe hypoxia than used in these experiments (<3 kPa) (Jiang et al. 1996). Hif1a immunoblotting was attempted to assess whether the transition from 20 to 4 kPa in CDmod conditions led to sustained increased expression of this important transcription factor. This proved technically difficult and we are unable to conclude whether Hif1a protein levels were altered under CDmod conditions. Interestingly, levels of a key cytoprotective, hypoxia‐responsive micro‐RNA, mir‐210, is increased to nearly threefold under CDmod conditions. Studies in myoblasts have confirmed marked induction of mir‐210 in severe hypoxia (1 kPa) as well as Hif1a‐dependent increased mir‐210 expression during differentiation even under normoxia (Cicchillitti et al. 2012). It is noteworthy that mir‐210 expression is not required for myogenesis or muscle regeneration but it does confer marked protection against oxidative stress (Cicchillitti et al. 2012) and acute muscle tissue ischemia (Zaccagnini et al. 2014). The cytoprotective properties of mir‐210 suggest higher expression of mir‐210 under CDmod conditions may be relevant to the observed relative resistance to atrophy‐inducing treatments such as Dex, compared with the effects of the same drug when used under STD conditions.

The increase in the number and uniformity of myotubes, the lower maturation index and high fusion index under CDmod conditions are strikingly different to STD conditions. These features are clearly advantageous for monitoring changes in morphology or gene expression under different experimental conditions. In particular, myotubes under STD conditions frequently develop very large myotubes/myosacs with local accumulations of nuclei (Fig. 1D) and a large proportion of reserve myoblasts are observed (Fig. 1E). Both these features may affect measures of global gene expression and responses to atrophy treatment. The presence of large myosacs, has a disproportionate impact on mean myotube diameter under STD conditions and may also skew measurements of myotube adaptations to atrophy treatments. The results of this study demonstrate that Dex treatment in STD conditions causes myotube atrophy (Table 2, Fig. 3A) and this is consistent with results of other authors who have used STD conditions to study molecular mechanisms of muscle wasting (Menconi et al. 2008). In contrast, under CDmod conditions Dex treatment for 72 h did not lead to any significant reduction in protein yield or myotube diameter (Table 2, Fig. 3A). However, it is not clear that the rapid, reproducible muscle loss observed in Dex‐induced experimental atrophy, accurately reflects the mechanisms of steroid myopathy observed in patients. Clinical muscle atrophy related to steroid use is idiosyncratic and more common in patients taking higher doses for long periods (Batchelor et al. 1997) despite the fact that most patients also have other underlying acute or chronic disease. Whilst the mechanisms and susceptibility factors for clinical steroid myopathy are still debated (Minetto et al. 2011), the differences in response to Dex treatment under CDmod conditions appears more consistent with the clinical response. Furthermore, despite this absence of myotube atrophy, Dex treatment did lead to upregulation of levels of mRNAs encoding Fbxo32 (56%) and MuRF1 (90%) (Table 2). These muscle‐specific ubiquitin E3 ligases are activated under conditions of rapid muscle atrophy (Lecker et al. 2004) and can contribute to net muscle proteolysis under certain conditions (Bodine et al. 2001). However, our results following Dex treatment under CDmod conditions suggest that their activation can form part of an adaptive response rather than indicating the presence of net muscle catabolism.

To investigate the use of CDmod culture conditions with a clinically relevant atrophy treatment we chose a combination of dexamethasone and doxorubicin. Importantly, dexamethasone is frequently administered with doxorubicin as an anti‐emetic (Herrstedt 2008) or as part of the combined anti‐cancer treatment (Pui and Evans 2006). Multi‐agent chemotherapy regimens, including anthracyclines like doxorubicin, may contribute to muscle loss and dysfunction (Scheede‐Bergdahl and Jagoe 2013) and other authors have shown that doxorubicin causes increased ROS production and atrophy in cultured C2C12 myotubes (Gilliam et al. 2012). Finally, in recent animal studies, we have shown that repeated cyclical administration of the combination of dexamethasone and doxorubicin (DD) leads to muscle loss and impaired mitochondrial respiration which persists even 3 months after all treatments had stopped (Gouspillou et al. 2015). Using DD treatment at clinically relevant concentrations, for example, doxorubicin 100 ng/mL or 0.2 *μ*mol/L (Barpe et al. 2010) in CDmod conditions, we found that, in contrast to Dex alone, DD caused atrophy of myotubes and significant reduction in Mb protein expression. Curcumin, a naturally occurring polyphenol which modulates several key regulatory signaling molecules (Shehzad and Lee 2013), partially prevented DD‐induced changes on myotube morphology, protein expression and disruption of the mitochondrial network (Table 3, Fig. 4). This is consistent with prior literature showing a skeletal muscle protective effects of curcumin in different forms of muscle injury (Avci et al. 2012) one model of tumor‐related muscle wasting (Siddiqui et al. 2009) and even chemotherapy (cisplatin) ‐induced mitochondrial dysfunction and oxidative stress in tissues such as liver, brain and kidney (Waseem and Parvez 2013; Waseem et al. 2013). However, in our experiments at clinically relevant concentrations (0.1 *μ*mol/L; 36.8 ng/mL) (Dhillon et al. 2008), curcumin had only modest protective effect on DD‐treated myotubes, and may not be sufficiently active to completely protect against chemotherapy‐related muscle dysfunction. Future studies may be warranted to explore the effect of combination with other potential myoprotective agents such as n‐3 polyunsaturated fatty acids (Murphy et al. 2011) or resveratrol (Shadfar et al. 2011).

Myotube growth, morphology and responses to atrophy treatments were altered when culture conditions were adjusted to better represent those found in vivo in patients with advanced cancer. The use of STD culture conditions may result in misleading conclusions when attempting to study mechanisms of muscle wasting in humans if atrophy responses are artificially amplified. In contrast, myotubes cultured under CDmod conditions appear more resistant to commonly used experimental atrophy treatments. The molecular mechanisms underlying the differences observed when myotubes are cultured under CDmod and STD conditions remain to be clarified more fully, but our data suggests that induction of mir‐210 expression under CDmod conditions may have an important protective role. Taken together the results support the case for more critical assessment of culture conditions and the type and doses of drugs used for in vivo models of muscle atrophy. It seems plausible that this approach will yield improvements in performance of preclinical tissue culture models to study molecular mechanisms of muscle atrophy and for testing and selection of potential myoprotective agents. Further efforts to optimize and establish a consensus on the use of physiologically relevant myotube culture conditions in clinically focused muscle research are warranted.

## Conflict of Interest

The authors declare that they have no conflict of interest.

## Supporting information




**Data S1.** Methodology and data for additional atrophy treatments.

## References

[phy213726-bib-0001] Antoun, S. , L. Birdsell , M. B. Sawyer , P. Venner , B. Escudier , and V. E. Baracos . 2010 Association of skeletal muscle wasting with treatment with sorafenib in patients with advanced renal cell carcinoma: results from a placebo‐controlled study. J. Clin. Oncol. 28:1054–1060. 20085939 10.1200/JCO.2009.24.9730

[phy213726-bib-0002] Avci, G. , H. Kadioglu , A. O. Sehirli , S. Bozkurt , O. Guclu , E. Arslan , et al. 2012 Curcumin protects against ischemia/reperfusion injury in rat skeletal muscle. J. Surg. Res. 172:e39–e46. 22079841 10.1016/j.jss.2011.08.021

[phy213726-bib-0003] Barpe, D. R. , D. D. Rosa , and P. E. Froehlich . 2010 Pharmacokinetic evaluation of doxorubicin plasma levels in normal and overweight patients with breast cancer and simulation of dose adjustment by different indexes of body mass. Eur. J. Pharm. Sci. 41:458–463. 20688160 10.1016/j.ejps.2010.07.015

[phy213726-bib-0004] Batchelor, T. T. , L. P. Taylor , H. T. Thaler , J. B. Posner , and L. M. DeAngelis . 1997 Steroid myopathy in cancer patients. Neurology 48:1234–1238. 9153449 10.1212/wnl.48.5.1234

[phy213726-bib-0005] Bayol, S. , C. Brownson , and P. T. Loughna . 2005 Electrical stimulation modulates IGF binding protein transcript levels in C2C12 myotubes. Cell Biochem. Funct. 23:361–365. 15584093 10.1002/cbf.1118

[phy213726-bib-0006] Blazejczyk, M. , M. Miron , and R. Nadon . 2007 FlexArray: a statistical data analysis software for gene expression microarrays. Genome Quebec, Canada.

[phy213726-bib-0007] Bodine, S. C. , E. Latres , S. Baumhueter , V. K. M. Lai , L. Nunez , B. A. Clarke , et al. 2001 Identification of ubiquitin ligases required for skeletal muscle atrophy. Science 294:1704–1708. 11679633 10.1126/science.1065874

[phy213726-bib-0008] Boonen, K. J. M. , D. W. J. Van Der Schaft , F. P. Baaijens , and M. J. Post . 2011 Interaction between electrical stimulation, protein coating and matrix elasticity: a complex effect on muscle fibre maturation. J. Regen. Med Tissue Eng. 5:60–68. 10.1002/term.289 20603881

[phy213726-bib-0009] Bossola, M. , M. Muscaritoli , R. Bellantone , F. Pacelli , A. Cascino , A. N. T. O. N. I. O. Sgadari , et al. 2000 Serum tumour necrosis factor‐alpha levels in cancer patients are discontinuous and correlate with weight loss. Eur. J. Clin. Invest. 30:1107–1112. 11122326 10.1046/j.1365-2362.2000.00751.x

[phy213726-bib-0010] Cicchillitti, L. , V. Di Stefano , E. Isaia , L. Crimaldi , P. Fasanaro , V. Ambrosino , et al. 2012 Hypoxia‐inducible factor 1‐*α* induces miR‐210 in normoxic differentiating myoblasts. J. Biol. Chem. 287:44761–44771. 23148210 10.1074/jbc.M112.421255 PMC3531789

[phy213726-bib-0011] Cooper, S. T. , A. L. Maxwell , E. Kizana , M. Ghoddusi , E. C. Hardeman , I. E. Alexander , et al. 2004 C2C12 co‐culture on a fibroblast substratum enables sustained survival of contractile, highly differentiated myotubes with peripheral nuclei and adult fast myosin expression. Cell Motil. Cytoskeleton 58:200–211. 15146538 10.1002/cm.20010

[phy213726-bib-0012] DeQuach, J. A. , V. Mezzano , A. Miglani , S. Lange , G. M. Keller , F. Sheikh , et al. 2010 Simple and high yielding method for preparing tissue specific extracellular matrix coatings for cell culture. PLoS ONE 5:e13039. 20885963 10.1371/journal.pone.0013039 PMC2946408

[phy213726-bib-0013] Dhillon, N. , B. B. Aggarwal , R. A. Newman , R. A. Wolff , A. B. Kunnumakkara , J. L. Abbruzzese , et al. 2008 Phase II trial of curcumin in patients with advanced pancreatic cancer. Clin. Cancer Res. 14:4491–4499. 18628464 10.1158/1078-0432.CCR-08-0024

[phy213726-bib-0014] Dobs, A. S. , R. V. Boccia , C. C. Croot , N. Y. Gabrail , J. T. Dalton , M. L. Hancock , et al. 2013 Effects of enobosarm on muscle wasting and physical function in patients with cancer: a double‐blind, randomised controlled phase 2 trial. Lancet Oncol. 14:335–345. 23499390 10.1016/S1470-2045(13)70055-X PMC4898053

[phy213726-bib-0015] Fearon, K. , F. Strasser , S. D. Anker , I. Bosaeus , E. Bruera , R. L. Fainsinger , et al. 2011 Definition and classification of cancer cachexia: an international consensus. Lancet Oncol. 12:489–495. 21296615 10.1016/S1470-2045(10)70218-7

[phy213726-bib-0016] Gilliam, L. A. , J. S. Moylan , E. W. Patterson , J. D. Smith , A. S. Wilson , Z. Rabbani , et al. 2012 Doxorubicin acts via mitochondrial ROS to stimulate catabolism in C2C12 myotubes. Am. J. Physiol. Cell Physiol. 302:C195–C202. 21940668 10.1152/ajpcell.00217.2011 PMC3328915

[phy213726-bib-0017] Gouspillou, G. , C. Scheede‐Bergdahl , S. Spendiff , M. Vuda , B. Meehan , H. Mlynarski , et al. 2015 Anthracycline‐containing chemotherapy causes long‐term impairment of mitochondrial respiration and increased reactive oxygen species release in skeletal muscle. Sci. Rep. 5:1–10. 10.1038/srep08717 PMC4346812 25732599

[phy213726-bib-0018] Green, G. A. , R. A. Darnall , T. B. Bierd , and J. M. Adams . 1989 Effect of aortic balloon inflation on ventilation and brain stem blood flow in piglets. J. Appl. Physiol. 66:2174–2180. 2501276 10.1152/jappl.1989.66.5.2174

[phy213726-bib-0019] Guttridge, D. C. , M. W. Mayo , L. V. Madrid , C. Y. Wang , and A. S. Jr Baldwin . 2000 NF‐kappaB‐induced loss of MyoD messenger RNA: possible role in muscle decay and cachexia. Science 289:2363–2366. 11009425 10.1126/science.289.5488.2363

[phy213726-bib-0020] Herrstedt, J. 2008 Antiemetics: an update and the MASCC guidelines applied in clinical practice. Nat. Clin. Pract. Oncol. 5:32–43. 18097455 10.1038/ncponc1021

[phy213726-bib-0021] Jiang, B. H. , G. L. Semenza , C. Bauer , and H. H. Marti . 1996 Hypoxia‐inducible factor 1 levels vary exponentially over a physiologically relevant range of O2 tension. Am. J. Physiol. 271:C1172–C1180. 8897823 10.1152/ajpcell.1996.271.4.C1172

[phy213726-bib-0022] Keeley, T. P. , R. C. Siow , R. Jacob , and G. E. Mann . 2017 A PP2A‐mediated feedback mechanism controls Ca2 + ‐dependent NO synthesis under physiological oxygen. FASEB J. 31:5172–5183. 28760745 10.1096/fj.201700211R PMC5690389

[phy213726-bib-0023] Kroehne, V. , I. Heschel , F. Schügner , D. Lasrich , J. W. Bartsch , and H. Jockusch . 2008 Use of a novel collagen matrix with oriented pore structure for muscle cell differentiation in cell culture and in grafts. J. Cell Mol. Med. 12:1640–1648. 18194451 10.1111/j.1582-4934.2008.00238.x PMC2680279

[phy213726-bib-0024] Langen, R. C. , A. M. Schols , M. C. Kelders , E. F. Wouters , and Y. M. Janssen‐Heininger . 2001 Inflammatory cytokines inhibit myogenic differentiation through activation of nuclear factor‐kappaB. FASEB J. 15:1169–1180. 11344085 10.1096/fj.00-0463

[phy213726-bib-0025] Langen, R. C. , A. M. Schols , M. C. Kelders , E. F. Wouters , and Y. M. Janssen‐Heininger . 2003 Enhanced myogenic differentiation by extracellular matrix is regulated at the early stages of myogenesis. In Vitro Cell. Dev. Biol. Anim. 39:163–169. 14505430 10.1007/s11626-003-0011-2

[phy213726-bib-0026] Lecker, S. H. , R. T. Jagoe , A. Gilbert , M. Gomes , V. Baracos , J. Bailey , et al. 2004 Multiple types of skeletal muscle atrophy involve a common program of changes in gene expression. FASEB J. 18:39–51. 14718385 10.1096/fj.03-0610com

[phy213726-bib-0027] Lerner, L. , T. G. Hayes , N. Tao , B. Krieger , B. Feng , Z. Wu , et al. 2015 Plasma growth differentiation factor 15 is associated with weight loss and mortality in cancer patients. J. Cachexia Sarcopenia Muscle 6:317–324. 26672741 10.1002/jcsm.12033 PMC4670740

[phy213726-bib-0028] Macdonald, N. 2007 Cancer symptom control trials: how may we advance this field? Curr. Oncol. 14:86–88. 17593980 10.3747/co.2007.116 PMC1899359

[phy213726-bib-0029] Marotta, M. , R. Bragos , and A. M. Gomez‐Foix . 2004 Design and performance of an electrical stimulator for long‐term contraction of cultured muscle cells. Biotechniques 36:68–73. 14740487 10.2144/04361ST01

[phy213726-bib-0030] Martin, N. R. , K. Aguilar‐Agon , G. P. Robinson , D. J. Player , M. C. Turner , S. D. Myers , et al. 2017 Hypoxia impairs muscle function and reduces myotube size in tissue engineered skeletal muscle. J. Cell. Biochem. 118:2599–2605. 28294416 10.1002/jcb.25982 PMC5518201

[phy213726-bib-0031] Martín, F. , F. Santolaria , N. Batista , A. Milena , E. González‐Reimers , M. J. Brito , et al. 1999 Cytokine levels (IL‐6 and IFN‐gamma), acute phase response and nutritional status as prognostic factors in lung cancer. Cytokine 11:80–86. 10080883 10.1006/cyto.1998.0398

[phy213726-bib-0032] Menconi, M. , P. Gonnella , V. Petkova , S. Lecker , and P. O. Hasselgren . 2008 Dexamethasone and corticosterone induce similar, but not identical, muscle wasting responses in cultured L6 and C2C12 myotubes. J. Cell. Biochem. 105:353–364. 18615595 10.1002/jcb.21833 PMC2901105

[phy213726-bib-0033] Minetto, M. A. , F. Lanfranco , G. Motta , S. Allasia , E. Arvat , and G. d'Antona . 2011 Steroid myopathy: some unresolved issues. J. Endocrinol. Invest. 34:370–375. 21677507 10.1007/BF03347462

[phy213726-bib-0034] Mozetic, P. , S. Maria Giannitelli , M. Gori , M. Trombetta , and A. Rainer . 2017 Engineering muscle cell alignment through 3D bioprinting. J. Biomed. Mater. Res. A 105:2582–2588. 28544472 10.1002/jbm.a.36117

[phy213726-bib-0035] Murphy, R. A. , M. Mourtzakis , Q. S. Chu , V. E. Baracos , T. Reiman , and V. C. Mazurak . 2011 Nutritional intervention with fish oil provides a benefit over standard of care for weight and skeletal muscle mass in patients with nonsmall cell lung cancer receiving chemotherapy. Cancer 117:1775–1782. 21360698 10.1002/cncr.25709

[phy213726-bib-0036] Pfaffl, M. W. , G. W. Horgan , and L. Dempfle . 2002 Relative expression software tool (REST) for group‐wise comparison and statistical analysis of relative expression results in real‐time PCR. Nucleic Acids Res. 30:1–10. 11972351 10.1093/nar/30.9.e36 PMC113859

[phy213726-bib-0037] Pisani, D. F. , and C. A. Dechesne . 2005 Skeletal muscle HIF‐1alpha expression is dependent on muscle fiber type. J. Gen. Physiol. 126:173–178. 16043777 10.1085/jgp.200509265 PMC2266573

[phy213726-bib-0038] Prado, C. M. , J. R. Lieffers , L. J. McCargar , T. Reiman , M. B. Sawyer , L. Martin , et al. 2008 Prevalence and clinical implications of sarcopenic obesity in patients with solid tumours of the respiratory and gastrointestinal tracts: a population‐based study. Lancet Oncol. 9:629–635. 18539529 10.1016/S1470-2045(08)70153-0

[phy213726-bib-0039] Prado, C. M. , V. E. Baracos , L. J. McCargar , T. Reiman , M. Mourtzakis , K. Tonkin , et al. 2009 Sarcopenia as a determinant of chemotherapy toxicity and time to tumor progression in metastatic breast cancer patients receiving capecitabine treatment. Clin. Cancer Res. 15:2920–2926. 19351764 10.1158/1078-0432.CCR-08-2242

[phy213726-bib-0040] Pui, C.‐H. , and W. E. Evans . 2006 Treatment of acute lymphoblastic leukemia. N. Engl. J. Med. 354:166–178. 16407512 10.1056/NEJMra052603

[phy213726-bib-0041] R Development Core Team . 2012 R: a language and environment for statistical computing. R Foundation for Statistical Computing, Vienna, Austria.

[phy213726-bib-0042] Rasband, W.S . (1997) ImageJ. US National Institutes of Health, Bethesda, Maryland, USA, http://imagej.nih.gov/ij/.

[phy213726-bib-0043] Richardson, R. S. , E. A. Noyszewski , K. F. Kendrick , J. S. Leigh , and P. D. Wagner . 1995 Myoglobin O2 desaturation during exercise. Evidence of limited O2 transport. J. Clin. Invest. 96:1916–1926. 7560083 10.1172/JCI118237 PMC185828

[phy213726-bib-0044] Sacheck, J. M. , J. P. K. Hyatt , A. Raffaello , R. T. Jagoe , R. R. Roy , V. R. Edgerton , et al. 2007 Rapid disuse and denervation atrophy involve transcriptional changes similar to those of muscle wasting during systemic diseases. FASEB J. 21:140–155. 17116744 10.1096/fj.06-6604com

[phy213726-bib-0045] van der Schaft, D. W. , A. C. van Spreeuwel , K. J. Boonen , M. L. Langelaan , C. V. Bouten , and F. P. Baaijens . 2013 Engineering skeletal muscle tissues from murine myoblast progenitor cells and application of electrical stimulation. J. Vis. Exp. e4267 https://doi.org/10.3791/4267 23542531 10.3791/4267 PMC3639551

[phy213726-bib-0046] Scheede‐Bergdahl, C. , and R. T. Jagoe . 2013 After the chemotherapy: potential mechanisms for chemotherapy‐induced delayed skeletal muscle dysfunction in survivors of acute lymphoblastic leukaemia in childhood. Front. Pharmacol. 4:49. 23626576 10.3389/fphar.2013.00049 PMC3630332

[phy213726-bib-0047] Sciancalepore, M. , T. Coslovich , P. Lorenzon , G. Ziraldo , and G. Taccola . 2015 Extracellular stimulation with human “noisy” electromyographic patterns facilitates myotube activity. J. Muscle Res. Cell Motil. 36:349–357. 26377755 10.1007/s10974-015-9424-2

[phy213726-bib-0048] Shadfar, S. , M. E. Couch , K. A. McKinney , L. J. Weinstein , X. Yin , J. E. Rodríguez , et al. 2011 Oral resveratrol therapy inhibits cancer‐induced skeletal muscle and cardiac atrophy in vivo. Nutr. Cancer 63:749–762. 21660860 10.1080/01635581.2011.563032 PMC3623008

[phy213726-bib-0049] Shehzad, A. , and Y. S. Lee . 2013 Molecular mechanisms of curcumin action: signal transduction. BioFactors 39:27–36. 23303697 10.1002/biof.1065

[phy213726-bib-0050] Siddiqui, R. A. , S. Hassan , K. A. Harvey , T. Rasool , T. Das , P. Mukerji , et al. 2009 Attenuation of proteolysis and muscle wasting by curcumin c3 complex in MAC16 colon tumour‐bearing mice. Br. J. Nutr. 102:967–975. 19393114 10.1017/S0007114509345250

[phy213726-bib-0051] Stewart Coats, A. J. , G. F. Ho , K. Prabhash , S. Haehling , J. Tilson , R. Brown , et al. 2016 Espindolol for the treatment and prevention of cachexia in patients with stage III/IV non‐small cell lung cancer or colorectal cancer: a randomized, double‐blind, placebo‐controlled, international multicentre phase II study (the ACT‐ONE trial). J. Cachexia Sarcopenia Muscle 7:355–365. 27386169 10.1002/jcsm.12126 PMC4929828

[phy213726-bib-0052] Temel, J. S. , A. P. Abernethy , D. C. Currow , J. Friend , E. M. Duus , Y. Yan , et al. 2016 Anamorelin in patients with non‐small‐cell lung cancer and cachexia (ROMANA 1 and ROMANA 2): results from two randomised, double‐blind, phase 3 trials. Lancet Oncol. 17:519–531. 26906526 10.1016/S1470-2045(15)00558-6

[phy213726-bib-0053] Thelen, M. H. , W. S. Simonides , and C. van Hardeveld . 1997 Electrical stimulation of C2C12 myotubes induces contractions and represses thyroid‐hormone‐dependent transcription of the fast‐type sarcoplasmic‐reticulum Ca2 + ‐ATPase gene. Biochem. J. 321:845–848. 9032474 10.1042/bj3210845 PMC1218143

[phy213726-bib-0054] Tusher, V. G. , R. Tibshirani , and G. Chu . 2001 Significance analysis of microarrays applied to the ionizing radiation response. Proc. Natl Acad. Sci. USA 98:5116–5121. 11309499 10.1073/pnas.091062498 PMC33173

[phy213726-bib-0055] Waseem, M. , and S. Parvez . 2013 Mitochondrial dysfunction mediated cisplatin induced toxicity: modulatory role of curcumin. Food Chem. Toxicol. 53:334–342. 23246825 10.1016/j.fct.2012.11.055

[phy213726-bib-0056] Waseem, M. , P. Kaushik , and S. Parvez . 2013 Mitochondria‐mediated mitigatory role of curcumin in cisplatin‐induced nephrotoxicity. Cell Biochem. Funct. 1:678–684. 10.1002/cbf.2955 23408677

[phy213726-bib-0057] Zaccagnini, G. , B. Maimone , V. Di Stefano , P. Fasanaro , S. Greco , A. Perfetti , et al. 2014 Hypoxia‐induced miR‐210 modulates tissue response to acute peripheral ischemia. Antioxid. Redox Signal. 21:1177–1188. 23931770 10.1089/ars.2013.5206 PMC4142832

