# Supplementary material for: Physiological culture conditions alter myotube morphology and responses to atrophy treatments: implications for in vitro research on muscle wasting
Source: Physiol Rep. 2018 Jun 21;6(12):e13726. doi: 10.14814/phy2.13726 (PMC6014447; doi:10.14814/phy2.13726)
Supplement: Supplementary file 1 — Data S1. Methodology and data for additional atrophy treatments. [file PHY2-6-e13726-s001.docx]

**Supplementary Data**:

Culturing myotubes in physiological conditions alters myotube morphology: implications for in vitro research on muscle wasting. Authors: Elodie Archer-Lahlou, Cathy Lan, R. Thomas Jagoe

Myoblast culture conditions:

For experiments comparing serum withdrawal, TNF-related weak inducer of apoptosis (TWEAK) and miRNA profiling myoblasts were seeded at 2.2 x 10^5^ cells per well and differentiated in medium containing 10% horse serum.

Additional atrophy-inducing treatments:

1) Serum withdrawal (Ser): myotubes were transferred to DM containing 0.5% horse serum on day 5 post-differentiation; 2) TWEAK treatment (Twk) (10 ng/mL TWEAK (R&D systems), supplemented with 100µM hydroxyurea).

miRNA expression profiling:

2µg total RNA from each experimental sample was covalently 3’-labeled with a single Hy3 (test) or Hy5 (reference) fluorophore per molecule, and co-hybridised using a fully-automated hybridisation station (Tecan HS400Pro) to microarrays pre-spotted in quadruplicate with capture probes for approximately 2000 mouse, rat, human sequences from miRBase (v.11 – v.16) (Hi-Power Labeling kit, Spike-in miRNA controls and miRCURY LNA microRNA arrays v.11, 5^th^ and 6^th^ generation, Exiqon, Woburn MA). To determine the variance for expression for each miRNA due to differential characteristics of the two fluorophores, the same image analysis steps was used for four ‘self-self’ experiments in which a single RNA sample was split between two aliquots, labeled with either Hy3 or Hy5 and hybridised to the same microarray. After hybridisation slides were scanned (Model G2505B, Agilent Technologies, Santa Clara, CA) and the two-colour images obtained were analysed using Spot v3.1 (CSIRO, NSW, Australia) package in R v2.12(1). Median signal intensity from each spot was corrected using subtraction of local background and within-array normalisation of the log2 ratio of the corrected spot signal from each channel was performed using the loess method in FlexArray v1.6.1 (2).

Statistical analysis of miRNA profiling:

For each experiment the mean normalized log2 expression ratios for each miRNA was recorded. No result was recorded if less than half of experimental replicates yielded analyzable results. Differential expression of COS vs STD was first determined using the Significance Analysis of Microarrays (SAM) v4.0 add-in for Microsoft Excel(3) on array-centred data from each of six experiments using a threshold false discovery rate of <1. For those genes identified using the SAM algorithm, a Student’s t-test was performed to compare gene-specific mean expression ratio in experimental samples, with mean ratio for same gene derived from self-self hybridization experiments. A t-test significance level was set as P<0.01 to achieve an expected false positive rate of less than 1. Only those genes which were positively identified as differentially expressed using both these methods, were used in further analysis.

1. R Development Core Team. R: A Language and Environment for Statistical Computing. Vienna, Austria: R Foundation for Statistical Computing; 2012

2. Blazejczyk M, Miron M, Nadon R. FlexArray: A statistical data analysis software for gene expression microarrays. Genome Quebec. 2007;Canada

3. Tusher VG, Tibshirani R, Chu G. Significance analysis of microarrays applied to the ionizing radiation response. Proc Natl Acad Sci USA. 2001;98:5116-5121.

**Supplemental Table 1**: The effect of modified culture conditions on myotube diameter and protein yield in presence of different atrophy-inducing treatments

|  |  | Myotube diameter (a.u.) | | | |  | Protein yield (mg/well) | | | | | |  |
| --- | --- | --- | --- | --- | --- | --- | --- | --- | --- | --- | --- | --- | --- |
| Model |  |  |  |  |  |  |  |  | |  |  | |  |
|  | Treatment | Untreated |  | Ser | Twk |  | Untreated |  | Ser | | | Twk | |
| STD | Raw | 28.1(2.6) |  | 18.9(2.6) | 21.2(0.7) |  | 754.4(118.2) |  | 426.0(53.7) | | | 634.5(54.1) | |
|  | Normalized (%) | 100 |  | 65.1(4.6) | 86.5(3.8) |  | 100 |  | 63.3 (3.8) | | | 90.0(3.6) | |
|  | Mean % change | -- |  | -34.9** | -13.5* |  | 0 |  | -36.7* | | | -10.0* | |
|  |  |  |  |  |  |  |  |  |  | | |  | |
| CDmod | Raw | 20.7(1.7) |  | 16.5(0.9) | 20.2(0.6) |  | 612.4(90.9) |  | 403.2(31.0) | | | 574.9(29.6) | |
|  | Normalized (%) | 73.4(4.3) |  | 61.8(2.1) | 82.3(5.1) |  | 80.3(4.0) |  | 59.7(1.5) | | | 81.7(1.5) | |
|  | Mean % change | -- |  | -15.9** | 12.1* |  | 0 |  | -25.7** | | | 1.7 | |

Notes: Culture conditions were exactly as described in Methods except that differentiation medium included 10% horse serum to amplify the effects of serum withdrawal. Atrophy treatments used were serum withdrawal (Ser) and TWEAK (Twk) treatments. Data presented as mean(SD) for raw data and normalized (%) to untreated myotubes under STD conditions for the same experimental replicate. Mean % change = mean difference between treated and untreated myotubes under the same culture conditions. For both Ser and Twk atrophy treatments the % reduction in diameter or protein yield was smaller in CDmod compared with STD culture conditions. Significance testing results indicated * P<0.05, ** P<0.001 comparing treated and untreated myotubes under same culture conditions for minimum of 3 independent experimental replicates.
